# Should We Open Fire on Microglia? Depletion Models as Tools to Elucidate Microglial Role in Health and Alzheimer’s Disease

**DOI:** 10.3390/ijms22189734

**Published:** 2021-09-08

**Authors:** Carmen Romero-Molina, Victoria Navarro, Sebastian Jimenez, Clara Muñoz-Castro, Maria V. Sanchez-Mico, Antonia Gutierrez, Javier Vitorica, Marisa Vizuete

**Affiliations:** 1 Departamento Bioquimica y Biologia Molecular, Facultad de Farmacia, Universidad de Sevilla, 41012 Seville, Spain; cromero15@us.es (C.R.-M.); vnavarro@us.es (V.N.); sejimu@gmail.com (S.J.); cmunoz5@us.es (C.M.-C.); marsanmic@gmail.com (M.V.S.-M.); 2 Instituto de Biomedicina de Sevilla (IBiS), Hospital Universitario Virgen del Rocio/CSIC/Universidad de Sevilla, 41012 Seville, Spain; 3 Centro de Investigacion Biomedica en Red Sobre Enfermedades Neurodegenerativas (CIBERNED), 28031 Madrid, Spain; agutierrez@uma.es; 4 Departamento Biologia Celular, Genetica y Fisiologia, Instituto de Investigacion Biomedica de Malaga (IBIMA), Facultad de Ciencias, Universidad de Malaga, 29071 Malaga, Spain

**Keywords:** microglia, Alzheimer’s disease, depletion, inflammation

## Abstract

Microglia play a critical role in both homeostasis and disease, displaying a wide variety in terms of density, functional markers and transcriptomic profiles along the different brain regions as well as under injury or pathological conditions, such as Alzheimer’s disease (AD). The generation of reliable models to study into a dysfunctional microglia context could provide new knowledge towards the contribution of these cells in AD. In this work, we included an overview of different microglial depletion approaches. We also reported unpublished data from our genetic microglial depletion model, *Cx3cr1^CreER^/Csf1r^flx/flx^*, in which we temporally controlled microglia depletion by either intraperitoneal (acute model) or oral (chronic model) tamoxifen administration. Our results reported a clear microglial repopulation, then pointing out that our model would mimic a context of microglial replacement instead of microglial dysfunction. Next, we evaluated the origin and pattern of microglial repopulation. Additionally, we also reviewed previous works assessing the effects of microglial depletion in the progression of Aβ and Tau pathologies, where controversial data are found, probably due to the heterogeneous and time-varying microglial phenotypes observed in AD. Despite that, microglial depletion represents a promising tool to assess microglial role in AD and design therapeutic strategies.

## 1. Microglia: Micro in Size but Macro in Functions, Highly Important in Alzheimer’s Disease

Microglia, the primary immune cells of the brain, not only survey the environment for pathogens and debris, but also play other important roles in the central nervous system (CNS), providing direct sustain to neurons and supporting myelinogenesis, synaptic plasticity, and the neoformation of vessels [1,2]. These glial cells account for 10–15% of the total cells in the adult CNS in humans [3] and 5–12% in mice [4]. Microglial cells derive from myeloid progenitors of the yolk sac that at embryonic day 8.5 colonize the mouse fetal brain, and actively proliferate at early postnatal days until reaching their definitive brain density [3]. Although the number of microglial cells remains constant during lifetime in mice and humans, a rapid turnover of microglia is maintained by a balanced coupling of microglial proliferation and apoptotic death [5]. In adult life, a 28% of microglia are renewed daily, meaning the lifespan for these cells 4.2 years [6]. Spatial heterogeneity of microglia has been observed in terms of density, functional markers and transcriptomic profiles. Moreover, microglia suffer transcriptional, morphological, and functional changes during aging, injury or pathological conditions, as multiple sclerosis, Parkinson’s disease (PD), and Alzheimer’s disease (AD), among others. 

In this review, we specifically focused on the role of microglia in AD. In patients, AD pathology develops along a continuum process (ATN), in which the amyloid deposition is considered the earlier event, preceding and triggering Tau pathology and neurodegeneration [7,8,9]. However, microglial role in the ATN continuum remains unsolved. Microglial activation and the loss of their homeostatic functions are considered as critical features in AD pathogenesis. Recent single-cell transcriptomic studies have identified different microglial subpopulations involved in AD [10,11,12,13,14], although the functional significance of this microglial diversity is not clearly understood. Moreover, microglial activation has been widely described in AD transgenic mice, but depending on the models, timing of pathology development and brain region, activated microglia can adopt a protective role or may acquire a cytotoxic phenotype, mediating neuronal damage. In amyloidogenic AD mouse models, a subset of activated microglial cells, named “disease-associated microglia” (DAM), cluster around amyloid plaques establishing a protective barrier [15,16]. This phenotype, characterized by the upregulation of genes involved in lysosomal, phagocytic, and lipid metabolic pathways, is ApoE-Trem2 dependent [15,16,17] and requires an oxidative metabolism [18]. Similar microglial transcriptomic profiles have also been described in several tauopathy models such as P301S and P301L mice [19,20]. However, microglial response is diverse in transgenic Tau models as, for instance, ThyTau22 mice manifest mild microglial activation, whereas P301S mice exhibit a strong microglial response [20]. Although the contribution of microglial cells to the progression and spread of pathogenic Tau species is still a matter of debate, it has recently been described that TREM2 loss of functions increases neuritic pathology and Tau spreading in amyloidogenic models [21].

Although microglial activation has been reported in several brain regions of AD patients [22,23,24], it is important to point out that, in the hippocampus, the microglial response is not as strong as reported for amyloidogenic mice and several Tau models [20,25]. Apart from the individual and regional heterogeneity, this apparent discordance between transgenic models and AD patients may be associated to the aging process itself, the main risk factor for late-onset AD, and/or to the chronic pathology of AD. Mouse models bearing familial AD mutations are frequently examined at relatively young ages compared to the elderly AD patients. Nevertheless, our results and others show that microglial activation increases with age in animals models with amyloid or Tau pathologies [20,26]. Then, other comorbidities present in AD patients as vascular deficiencies, hypertension, inflammatory diseases, obesity or diabetes mellitus could be involved in this distinct microglial response. What is more, transcriptomic studies from purified microglia showed few overlaps in differentially expressed genes during aging between humans and mice, hinting that microglia may age differently in both species [27].

Several evidences point to the decline of microglial defensive functions in AD. In this sense, we have described a microglial degenerative process in the hippocampus of AD patients, mainly mediated by pathological Tau species [25,28]. Other works have demonstrated how some variants in microglial specific genes, such as *Trem2* or *Cd33*, may alter microglial survival and function [29]. In the same line, a reduction in oxygen availability due to vascular alterations—typical of AD brains—may compromise microglial oxidative metabolism and, consequently, microglial activation [18]. In addition, we have reported that soluble phospho-Tau species may be toxic for microglial cells in vitro [20,28]. In short, all these factors may contribute to microglial dysfunction. However, it is still a matter of debate whether this phenomenon is a cause or a consequence of the typical AD pathological hallmarks. In view of this, it is essential to generate reliable mouse models mimicking the human defective microglial phenotype [25,30] to study the progression of Aβ and Tau pathologies in a context of microglial dysfunction. 

In the last years, microglial depletion mouse models have provided new insights into the role of these cells in physiological and pathological conditions. Here, we review different mouse models of microglial depletion, both in health and in AD, evaluating how reliable they could be as tools to study a context of microglial dysfunction or a context of microglial renewal. We recapitulate previous published data on the main microglial depletion strategies and, importantly, we also include unpublished data from our recently developed mouse model of conditional microglia depletion (*Cx3cr1^CreER^/Csf1r^flx/flx^*). We also comment on the origin and pattern of microglial repopulation process. Additionally, we review previous works in regards to the effects of microglial depletion and repopulation in the progression of Aβ and Tau pathologies. Outcomes are diverse and sometimes contradictory, but they open new research lines regarding the mechanisms underlying microglial proliferation and migration capabilities. Selective ablation of harmful microglia within suitable time windows and their replacement by protective microglia may be a promising therapeutic strategy for AD and other neurodegenerative diseases. 

## 2. Pharmacological and Genetic Microglial Depletion Models

Microglial viability and proliferation depend on signaling through the colony-stimulating factor1 receptor (CSF1R) [5,31,32,33] that belongs to the type III tyrosine kinase family, and is activated by two different cytokine ligands, colony stimulating factor-1 (CSF1) and interleukin-34 (IL-34) [34,35,36]. However, *Csf1r* is expressed on all myeloid cells [37,38], so the signaling interference through this receptor will not only affect microglial cells, but also peripheral macrophages, probably mediating an immunosuppressive effect. As it is widely known, *Csf1r* knock-out (KO) mice do not reach adult stage [3,39] so the suppression of this receptor should be carried out in adulthood, either through the administration of pharmacological inhibitors or through controlled genetic systems. As previously revised, different approaches give rise to variable depletion percentages, also dependent on the dose and length of treatments [40,41].

The first pharmacological approach trying to deplete microglial populations used a bisphosphonate drug, clodronate, packed in liposomes (Clo-Lip), which is rapidly taken up by phagocytic cells inducing their apoptotic death. Clo-Lip does not cross the blood–brain barrier (BBB), and consequently, needs to be administered by either intraparenchymal or intraventricular injection. Intraparenchymal Clo-Lip injection depletes between 30 and 60% of microglia 24 to 72 h after injection, but also produces astrocytic activation, releases proinflammatory cytokine and alters blood vessel integrity (reviewed in [42]). A better pharmacological strategy for microglial elimination was achieved by highly potent CSF1R tyrosine kinase inhibitors as PLX3397 and PLX647 that, after crossing the BBB, lead to microglia depletion without consequent inflammation, cytokine storm, or BBB damage, and no negative effects on mice behavior and cognition [43]. Depending on the dose and inhibitor used, different degrees of microglia depletion were reached and maintained throughout the treatment. Additional specific CSF1R inhibitors as JNJ-40346527, GW2580 and BLZ945 are available, and different studies have shown their dose-dependent effects on microglial number and phenotype [43,44]. Recently, a new and highly specific inhibitor for CSF1R, PLX5622, has been developed, improving BBB penetrance compared to PLX3397 [45]. However, and unexpectedly, the effect of these small CSF1R inhibitors is not restricted to microglia, but also affects the whole macrophage population and hematopoiesis [46]. Moreover, all these inhibitors are not specific for CSF1R, as they also inhibit three other kinases as FLT3, PDGFR, and KIT [47] and leads to broad myelosuppression, affecting macrophages, osteoclasts, and mast cells, among other cells. Additionally, it should be considered that PLX treatments may have a detrimental effect on neurons as CSF1R signaling has been demonstrated to enhance neuronal survival [48]. Actually, Shi et al. showed that PLX3397 inhibit neurite outgrowth and mildly reduce neuron number in vitro [49]. Further experimental approaches are still necessary in order to specifically deplete microglia using these small CSF1R inhibitors without affecting other cell types or tissues.

A more selective microglial depletion, with little effects on peripheral tissues, can be achieved by genetic manipulations based on the combination of cell type specific promoters coupled to suicide genes [50]. The initial approach was based on the expression of the suicide herpes simplex virus thymidine kinase (*HSV-1 TK*) transgene under the *Cd11b* promoter [51]. The administration of ganciclovir to *Cd11b-TK* mutant mice induces apoptosis of microglia, but also of CD11b+ bone marrow cells. To avoid myelotoxicity and the consequent mouse death, it is mandatory to combine this model with a bone-marrow chimera system, or alternatively administrate ganciclovir intraventricularly. Other genetic approaches to deplete myeloid population used diphtheria toxin (DT)-based models, in which myeloid promoter-driven Cre recombinase mouse lines (*Cx3cr1^Cre^*) were crossed with transgenic mice harboring genes for diphtheria toxin receptor (DTR) downstream of loxP-flanked STOP sequences. In this model, the administration of DT originated the acute cell death of all myeloid cells expressing DTR [52], although reached only short-lived depletion, less than 5 days.

On the other hand, the inducible *Cx3cr1^CreER^* line allows the targeting of microglia in a cell-type-specific and tamoxifen inducible fashion [53]. Two main *Cx3cr1^CreERT2^* inducible lines were created separately in which a tamoxifen-inducible Cre-recombinase is expressed under the control of the *Cx3cr1* promoter: *Cx3cr1^CreER/+:R26iDT-A/+^* and *Cx3cr1^CreER/+:R26iDTR/+^*. When activated by tamoxifen, nuclear translocation of the CreER fusion protein is transient and recombination occurs only for a limited period, so only long-lived cells as microglia, but not peripheral macrophages with a shorter lifespan, will be depleted. Later, the generation of conditional knockout mice harboring a loxP-flanked exon within the *Csf1r* gene (*Csf1r^flx/flx^*) has allowed spatial and temporal control of microglia upon combination with the appropriate Cre lines [54]. Additionally, the targeting of a more specific microglia-signature gene, as *Tmem119*, has allowed the generation of *Tmem119^CreERT2^* lines [55]. These new genetic models considerably represent an improvement in the manipulation of microglia providing a valuable tool for the functional study of these cells (reviewed in [40,41]).

## 3. Characterization of *Cx3cr1^CreER^/Csf1r^flx/flx^* Mice. Is It a Good Approach to Study Microglial Dysfunction?

Following the development of microglial depletion models and given the above-mentioned need of studying the role of microglia in AD progression, we and others aimed to study Aβ and Tau pathologies in AD microglia-depleted mice. Specifically, it will be of interest to mimic a context of microglial dysfunction, previously observed in the AD brain [28]. However, achieving significantly reduced levels of microglia for long periods is still a challenge in the field. 

**Figure 1:**
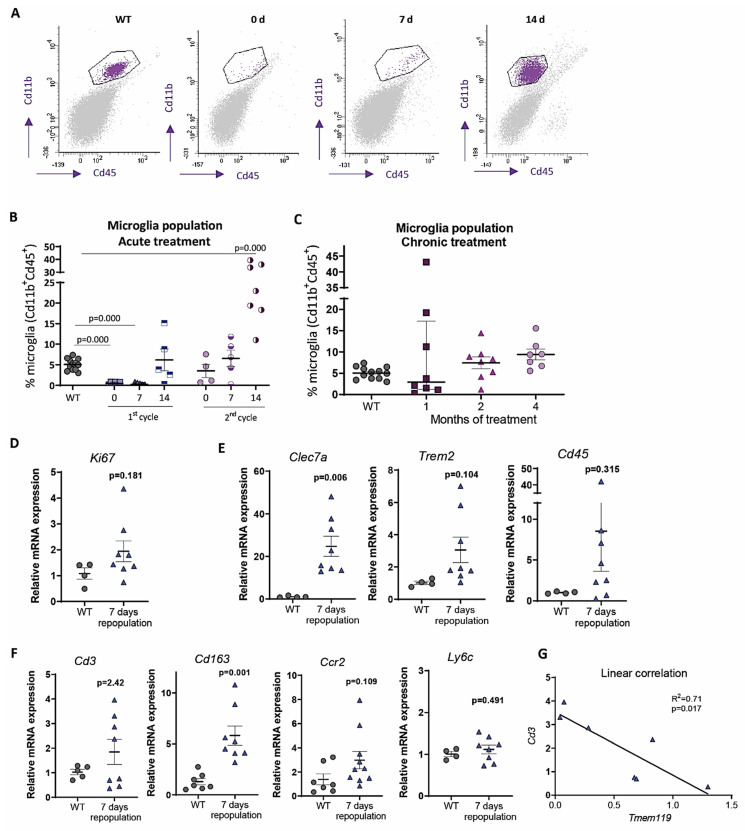
Microglial depletion is followed by a rapid repopulation, mediated by microglial activation, in 2-month-old *Cx3cr1^CreER^/Csf1r^flx/flx^* mice. (**A**). Representative flow cytometry images for the analysis of microglial cells (Cd45+Cd11b+) in WT and *Cx3cr1^CreER^/Csf1r^flx/flx^* mice subjected to acute tamoxifen treatment (7 days) and sacrificed 0, 7, or 14 days after the end of the treatment. (**B**). Quantification of microglial population after tamoxifen acute treatment, by flow cytometry, in cortical regions. The 1st cycle: 7 days tamoxifen; 2nd cycle: 7 days of tamoxifen administration after 14 days of the end of the first cycle. Mice were sacrificed 0, 7, or 14 days after the end of the corresponding cycle. (**C**). Microglial population, quantified by flow cytometry, in WT and *Cx3cr1^CreER^/Csf1r^flx/flx^* mice subjected to oral tamoxifen administration for 1, 2 or 4 months. Animals were sacrificed at the end of the treatment. (**D**–**F**). Quantification, by qPCR, of mRNA expression of the proliferation marker *Ki67*, normalized by *Gapdh* (D); microglial activation markers *Clec7a*, *Trem2*, *Cd45*, normalized by *Tmem119* (**E**); and immune infiltration markers *Cd3*, *Cd163*, *Ccr2* and *Ly6c*, normalized by *Gapdh* (**F**); in the hippocampus of WT and *Cx3cr1^CreER^/Csf1r^flx/flx^* mice subjected to acute tamoxifen treatment and sacrificed 7 days after. (**G**). Correlation between *Tmem119* (microglial homeostatic marker) and *Cd3* (lymphocytic marker) mRNA expression, quantified by qPCR and normalized by *Gapdh*, in the hippocampus of *Cx3cr1^CreER^/Csf1r^flx/flx^* mice subjected to acute tamoxifen treatment and sacrificed 7 days after the end of the treatment. See Supplementary Table S1 for Antibodies, Probes and Methods used. Statistical significance was analyzed using the *t*-test or the ANOVA test, followed by the Fisher LSD test.

In our research group, we have generated *Cx3cr1^CreER^/Csf1r^flx/flx^* mice by crossing *Cx3cr1^CreER^* × *Csf1r^flx/flx^* mice (both from Jackson laboratories) and we have firstly characterized them, in order to cross them with mice bearing Aβ and/or Tau pathology thereafter. By intraperitoneal tamoxifen injections (75 mg/kg for 7 days), we have achieved a 98% microglia depletion (Figure 1A,B), a similar percentage to the one achieved in previous published studies that used high PLXs doses (1200 mg/kg of pellet) [42]. The rapidity (7 days) of the depletion leaves no doubt that it is due to microglial death and not to an inhibition of proliferation. Furthermore, Elmore et al. (2014) showed that after CX3CR1 inhibition, not only is there no dedifferentiation of microglia towards other cell types, but apoptosis, through caspase 3 activation, seems to be the main mechanism underlying microglial death [32]. Additionally, Spangenberg et al. (2016) demonstrated, by using mice that constitutively express YFP under the *Rosa26* locus in all CSF1R expressing cells, that microglia are being eliminated and not simply downregulating their expression of specific markers [56]. 

Our results, in accordance with other reports [32,52,57], showed that acute microglial depletion (obtained after 7 days of tamoxifen treatment) is followed by a rapid and complete repopulation, reached 14 days after the end of the treatment; similar to PLX treatments [58]. Once the efficacy of acute depletion in our *Cx3cr1^CreER^/Csf1r^flx/flx^* model was confirmed, we planned to perform several cycles of acute treatments in order to analyze the repopulation capacity of microglia. Our final goal was to exhaust this proliferative capacity in order to achieve long-term sustained low microglial levels. Therefore, we evaluated microglial repopulation capacity in *Cx3cr1^CreER^/Csf1r^flx/flx^* mice after a second cycle of acute tamoxifen treatment. For that, after completing the first cycle of injections, we waited 14 days for recovery and again injected tamoxifen for 7 days. Flow cytometric quantification of microglial cells at the end of the second cycle indicated that microglial depletion was lower than that occurring in the first cycle (Figure 1B). This may be due either to the fact that we start from a slightly higher number of microglial cells (the percentage of microglial cells on day 14 of recovery after the first cycle is scarcely higher than the WT condition), and/or to the proliferation of a CSF1R-independent microglial population. Then, 14 days after the end of the second tamoxifen cycle, we observed a more pronounced peak of repopulation compared to the first cycle, revealing that a second depletion treatment did not dwindle microglial proliferation capacity. Moreover, a second tamoxifen-injection cycle increased mouse lethality up to 28% (data not shown), which prevented us from prolonged intraperitoneal treatments. 

In general, due to different reasons, few models achieve full chronic microglial depletion. In the *Cx3cr1^CreER^/Csf1r^flx/flx^* model, as DT administration is required, treatment is limited to acute periods (5 days) [52,59]. In the same line, the *Cd11b-TK* model allows a constrained microglial depletion (approximately up to 4 weeks) since ganciclovir administration induces BBB damage and myelotoxicity [60]. Likewise, the use of clodronate liposomes is not valid for long-term depletion since, due to its short half-life and BBB impermeability, it must be administered by intraparenchymal injections [61]. Then, the use of PLXs may be the most successful option, although microglia depletion only reaches a 30–50% in most of cases [62,63,64,65]. On the other hand, Zhu et al. (2020) have achieved a 60% of microglial depletion for 3 months by using the *Cx3cr1^Cre/R26DTA^* model and tamoxifen chow [66]. Most of authors associated all changes observed after depletion treatment to the absence of microglia, but it should be kept in mind that there was still a 40 to 70% of non-depleted microglia by using these approaches. Then, it should be considered that observed effects might also be due to changes in remaining microglia.

As it is widely known, Aβ and Tau pathologies, unlike stroke or acute injury, are associated to slow progressive diseases, which would require a longer microglia depletion in order to study microglial contribution. To this end, and in order to avoid the high toxicity of repeated acute tamoxifen cycles, we performed chronic oral tamoxifen administration (an estimated ingested dose of 40 mg/kg per mice; 250 mg/kg of tamoxifen in chow, Envigo), in our *Cx3cr1^CreER^/Csf1r^flx/flx^* mouse model. As shown in Figure 1C, this treatment was insufficient to maintain low levels of microglia. The 1-month tamoxifen treatment led to a non-significant reduction of microglial population (with high sex-independent variability), but after 2 and 4 months of treatment, microglial levels reached WT values. Likely, a proliferation of CSF1R-independent cells was taking place, as it will be lately discussed. Although we could consider combining tamoxifen treatments with specific proliferation inhibitors, it is still possible that there is a repopulation mediated by infiltration of peripheral immune cells. 

To summarize, thanks to the high microglial depletion obtained by acute tamoxifen treatment in our *Cx3cr1^CreER^/Csf1r^flx/flx^* model, we can assess that observed microglia after 14 days of repopulation correspond to an emerging population, in contrast to chronic models where remaining and emerging microglia constantly coexist. However, we failed in our attempt to achieve a maintained microglial depletion, and given the results obtained in chronic tamoxifen treatment, we consider that our model is not suitable to evaluate an environment of microglial degeneration, senescent or dysfunction. In contrast, it may be an appropriate model to study a context of continuous microglial replacement. 

## 4. Origin and Pattern of Microglial Repopulation

Assessing the efficacy of emerging microglia will contribute to validate the potential success of microglial replacement therapies. To this end, we focused on the characterization of the origin and pattern of microglial repopulation. We compared unpublished data from our *Cx3cr1^CreER^/Csf1r^flx/flx^* model to previous works, and we contemplated different possibilities such as microglial proliferation and/or peripheral immune infiltration. As previously mentioned, we (Figure 1A) and others [67] have described a pronounced microglial repopulation process after selective microglial depletion. In our *Cx3cr1^CreER^/Csf1r^flx/flx^* model, we observed that, 14 days after tamoxifen retrieval, microglial levels were similar to WT animals. More important, in spite of feeding *Cx3cr1^CreER^/Csf1r^flx/flx^* mice with tamoxifen for several months, microglial levels were similar to WT, suggesting that a continuous microglial replacement is taking place.

The origin of repopulating cells has not been unequivocally determined yet. Elmore et al. (2014) proposed that after microglial depletion, local progenitor microglial cells (nestin^+^) proliferated [32], while Bruttger et al. (2015) supported that remaining microglial cells increased their proliferative rate themselves [52]. Recent mapping studies, single cell sequencing analysis, and parabiosis experiments supported this second mechanism [5,68,69,70]. Our results also trend towards this hypothesis since we observed (Figure 1B) that the lower the depletion, the greater the repopulation. When *Cx3cr1^CreER^/Csf1r^flx/flx^* mice were subjected to a second cycle of intraperitoneal tamoxifen, microglial percentage at 0 days of repopulation is higher than after first cycle, and this correlated with a more pronounced microglial repopulation peak. Therefore, if there are more remaining microglia that can proliferate, repopulation is faster and more forceful. Konishi et al. (2020) have identified the expression of *Ki67* in microglia after depletion treatments [69], and we also observed a non-statistically significant increase in *Ki67* mRNA expression in *Cx3cr1^CreER^/Csf1r^flx/flx^* hippocampus after 7 days of repopulation (Figure 1D).

In addition, we observed a significant increment in the expression of the microglial activation marker *Clec7a* and a trend of increase in *Trem2* and *Cd45* markers after 7 days of repopulation (Figure 1E), which may allow the remaining microglia to be less dependent on CSF1R signaling. In this sense, Lodder et al. (2021) have shown that active Aβ-associated microglia were more resistant to PLX3397 treatment [71]. Additionally, it has been described in vitro that TREM2 signaling induces the activation of Syk pathway (downstream to CSF1R), in such a way that it synergizes with the CSF1-CSF1R axis [72]. Therefore, the increased expression of *Trem2* in the remaining/repopulating microglia could favor their tolerance to tamoxifen and would explain the moderate microglial levels recorded during chronic treatments. Furthermore, Zhan et al. (2020) have described that there is a MAC2^+^ microglial subpopulation, sharing similarities with microglial progenitors from the yolk sac and with immature microglia in early embryos, which survive to CSF1R inhibition and mediate microglia repopulation [73]. 

On the other hand, we may consider that niche repopulation may also be mediated through infiltration of peripheral immune cells. The fact that CNS-associated macrophages, monocyte-derived cells, and microglia display general macrophage markers hinders the differential study of these populations. Moreover, although macrophages remain transcriptomically different, they can acquire a microglial identity (such as morphology and clonal expansion) after being exposed to CNS environment [74,75]. Monocyte infiltration has been described in *Cd11b-TK* [76] and *Cx3cr1^CreER+^/R26^DTA+^* depletion models [77]. In fact, Lund et al. (2018) demonstrated that the transcriptomic signature of infiltrated cells (F4/80^hi^) [77] did not coincide with that of the local microglia characterized by Bennett et al. (2018) [63]. Regarding CSF1R inhibition models, in studies using PLXs, no changes have been reported in infiltration markers such as *Ccr2* [32], while in the *Cx3cr1^CreER^/Csf1r^flx/flx^* model, macrophage infiltration has been observed when microglial self-renewal is prevented [54]. In this sense, microglia could be responsible for the maintenance of CNS immune privilege in pathological situations [78].

As shown in Figure 1F, our data revealed a non-significant increment of *Cd3* expression in the hippocampus of *Cx3cr1^CreER^/Csf1r^flx/flx^* mice during repopulation compared to WT, which may suggest lymphocytic infiltration. Additionally, as higher microglia depletion (lower expression of *Tmem119* marker), higher *Cd3* expression (Figure 1G), supporting microglial role in avoiding peripheral immune cells infiltration. On the other hand, we reported a statistically significant increment of *Cd163* expression. Although in post-mortem tissue from AD patients (Muñoz-Castro et al., manuscript in preparation) and in some animal models [79,80], CD163 seems to be associated with CNS-associated macrophages and infiltration of monocytes-derived cells, in APP and TAU P301S mouse models we have not observed a clear infiltration of CD163^+^ cells in brain parenchyma (data not shown). In fact, other authors postulated that certain microglial subpopulations may express CD163 [1,81]. Taking into account that there was not an increment in the expression of the monocytic markers *Ccr2* or *Ly6c* (Figure 1F), the significant increase in *Cd163* observed after 7 days of repopulation could be associated to a microglial subpopulation. Thus, we postulate that microglia repopulation may be mainly mediated by microglial self-renewing, although the mechanisms and molecular events mediating it have not been clarified so far. It has been proposed that signaling through Il-1β [52], NF-KB [70] and Lgals3 [73] could be critical factors in microglial repopulation.

**Figure 2:**
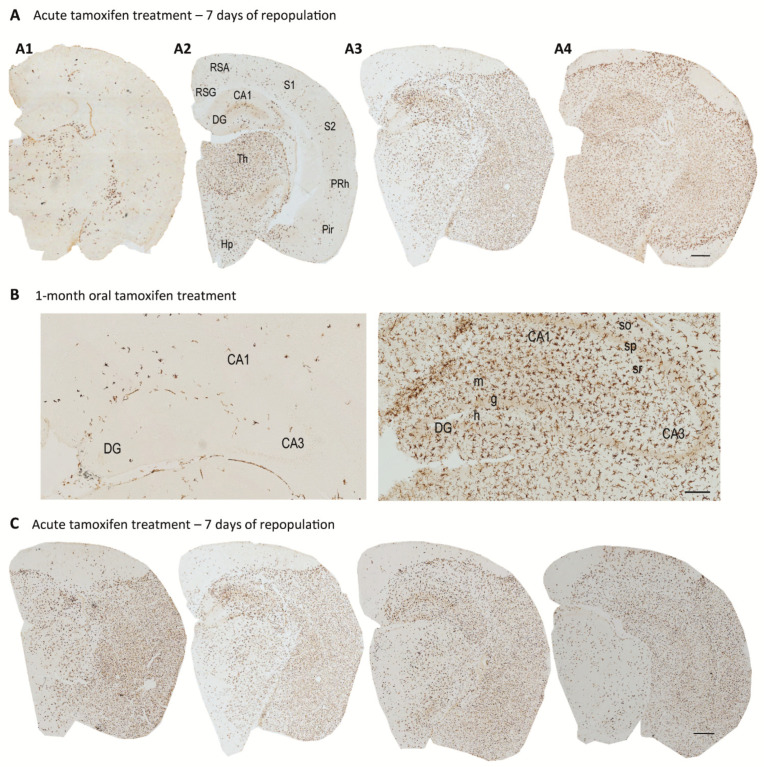
Microglial repopulation in *Cx3cr1^CreER^/Csf1r^flx/flx^* mice. (**A**,**B**). Microglial immunostaining (Iba1+ cells) in brain sections of *Cx3cr1^CreER^/Csf1r^flx/flx^* mice subjected (**A**) to acute tamoxifen administration (7 days) and sacrificed 7 days after the end of the treatment (*n* = 4 animals); (**B**) oral tamoxifen treatment for 1-month (*n* = 2 animals). (**C**). Consecutive brain sections immunostained with Iba1+ from a *Cx3cr1^CreER^/Csf1r^flx/flx^* mouse subjected to 7-days tamoxifen treatment and sacrificed 7 days after the end of the treatment. See Supplementary Table S1 for Antibody and Method used. RSA, retrosplenial agranular cortex; RSG, retrosplenial granular cortex; PRh, perirhinal cortex; Pir, piriform cortex; S1 primary somatosensory cortex; S2 secondary somatosensory cortex; Th, thalamus; Hp, hypothalamus; CA1, field CA1 of hippocampus; CA3, field CA3 of hippocampus; DG, dentate gyrus; so, stratum oriens; sp, stratum pyramidale; sr, stratum radiatum; m, molecular layer; g, granular layer; h, hilus. Scale bars: (**A**,**C**) 50 mm; B 200 μm.

Intriguingly, as shown in Figure 2, we observed a high variability in microglial levels during the repopulation process after acute and chronic (1-month) tamoxifen treatment. In Figure 2A, we showed four different *Cx3cr1^CreER^/Csf1r^flx/flx^* mice sacrificed 7 days after tamoxifen acute administration, which displayed important differences in microglial (Iba1^+^) staining. Similarly, in Figure 2B, we compared the hippocampus of two female *Cx3cr1^CreER^/Csf1r^flx/flx^* mice subjected to 1-month tamoxifen treatment; they showed opposite microglial levels. So far, we have not been able to associate this variability to any factor, as they were same-age animals and there was not a sex-correlation. As far as we know, a high variability between animals has not previously been reported when using other depletion models. As an exception, Shi et al. (2019) reported higher levels of microglial reduction in male compared to female mice using PLX3397, associated with reduced PLX3397 levels in the plasma [49]. Our genetic microglia depletion model avoids variable PLXs pharmacokinetics, but different tamoxifen CNS bioavailability should be considered. At the same time, there may be differences in CreER recombination efficiency. Microglial cells that do not undergo CSF1R ablation at the beginning of the treatment may proliferate, get more active and, then, become independent of CSF1R signaling. Consequently, they would be resistant to future tamoxifen administration and contribute to a higher repopulation. In short, the marked variability made us question the validity of our model and prevented us from obtaining consistent data. New efforts should be made to determine the mechanism underlying this shifting repopulation degree.

On the other hand, our results revealed a peculiar pattern of repopulation, with some areas totally depopulated compared to full-replenished regions (Figure 2A,C). A high variability in the repopulation pattern was again detected among mice, with some of them showing microglia cells mainly in the thalamus (Figure 2(A2)) while others displayed a more pronounced microglia replenishment in the cerebral cortex (Figure 2(A3)). Additionally, as shown in Figure 2(A4), there were some totally microglia-empty cortex portions, with a marked boundary separating them from fully-replenished cortex regions. More surprisingly, a rostro-caudal dynamic microglia repopulation pattern was detected in sequential immunostained sections from the same animal (Figure 2C). Varvel et al. (2012) reported a similar repopulation pattern in *Cd11b-TK* transgenic mice one week after 90% of microglia depletion [76]. However, in this study, authors showed that blood-derived monocytes were responsible of CNS repopulation. 

Casali et al. (2020) observed a variation in the percentage of microglia depletion across regions, achieving a 30% microglia depletion in subiculum, a 50% in hippocampus, and a 70% in cortical and thalamic regions in 5xFAD mice after 28 days of PLX5622 treatment [62]. In contrast, Spangenberg et al. (2019) reported more than a 99% microglia depletion in cortex after 10 weeks of PLX5622 in WT and 5xFAD mice, but a fraction of cells remained in the thalamus [45]. Up to date, we have no clear explanation for our striking repopulation pattern. However, Zhan et al. (2019) demonstrated, by dual-color labelling, that newborn microglia recolonized the parenchyma by forming distinctive clusters that maintained stable territorial boundaries over time, with minimal migratory diffusion [70]. It is possible that this regional heterogeneity in microglia repopulation could be related to a distinct origin of microglial precursors in each brain region during development [82]. Therefore, microglia from each brain region might show different rates of cell proliferation. In addition, the space and time pattern of microglia repopulation in the cerebral cortex recall how microglia colonize the developing neocortex, invading first the deeper layers and then progressing to the upper ones [83].

In short, although further work is required, the most supported hypothesis for microglial repopulation is microglia self-renewal, with a minor contribution of peripheral immune cells. In a novel way, our results open new key research lines in regards to addressing the mechanism underlying the variability in the degree and pattern of repopulation, which may shed light into our understanding of microglia proliferation and migration capabilities. 

## 5. Microglial Depletion as a Model of Microglial Replacement

Due to high mouse microglial proliferation capacity, these depletion models do not mimic a situation of microglial degeneration, as previously desired, but a context of microglial renewal. The characterization of emerging microglia’s capabilities will allow us to validate the efficacy of microglial depletion-repopulation strategies as potential therapeutic tools. This approach will be beneficial when microglia are hyperactive as well as in a context of microglial degeneration [28] or senescent [84], because in both cases microglia may contribute to neuron toxicity. As microglial chronic activation is sustained by an oxidative metabolism [18], this may compromise oxygen availability for other cellular populations. Additionally, an excessive microgliosis may induce microglial mitochondrial damage and be a major source of reactive oxidative species [85], leading to oxidative damage in neurons, to astrocyte reactivity [86] and to an exacerbation of the inflammatory cascade.

Repopulating microglia appear to fulfil functions of resident microglia and is capable of monitoring the environment and responding to acute stimuli [58,76,87]. Adult newborn microglia have been described to gradually regain steady-state maturity, transcriptionally clustering close to control microglia 2 weeks after depletion [70]. Zhan et al. (2019) also showed that the restoration of microglial homeostatic density requires NF-κB signaling as well as apoptotic egress of excessive cells [70]. In accordance, Huang et al. (2018) found no transcriptomic differences in repopulated microglia (2-month after depletion treatment) compared to resident microglia, neither in resting conditions nor after LPS challenge [68]. More recently, Gratuze et al. (2021) also described a homeostatic gene signature and equal ability to cluster around amyloid deposits in repopulated microglia in an AD mouse model [88].

**Figure 3:**
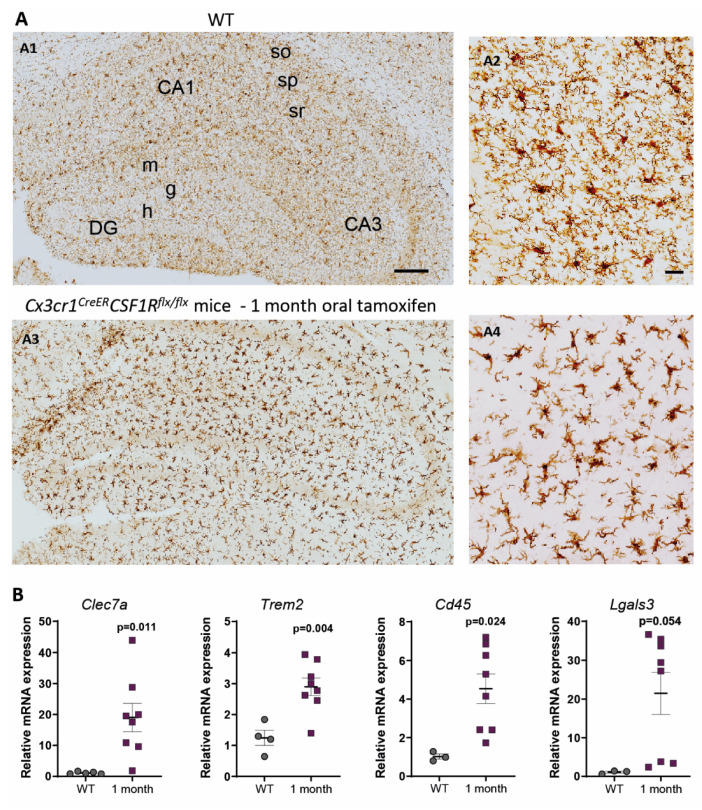
Microglial activation after 1-month oral tamoxifen administration in *Cx3cr1^CreER^/Csf1r^flx/flx^* mice. (**A**). Iba1 immunostaining in WT and *Cx3cr1^CreER^/Csf1r^flx/flx^* mice subjected to 1-month of tamoxifen treatment. CA1, field CA1 of hippocampus; CA3, field CA3 of hippocampus; DG, dentate gyrus; so, stratum oriens; sp, stratum pyramidale; sr, stratum radiatum; m, molecular layer; g, granular layer; h, hilus. Scale bars: A1, A3 200 μm; A2, A4 10 μm. (**B**). mRNA expression of microglial activation markers (*Clec7a*, *Trem2*, *Cd45* and *Lgals3*), quantified by qPCR and normalized by *Tmem119* (microglial homeostatic marker), in the hippocampus of WT and *Cx3cr1^CreER^/Csf1r^flx/flx^* mice treated with tamoxifen for 1 month. See Supplementary Table S1 for Antibody, Probes and Methods used. Statistical significance was analyzed using the *t*-test.

In contrast, under 1-month continuous tamoxifen treatment in our *Cx3cr1^CreER^/Csf1r^flx/flx^* model, we observed that emerging microglia displayed an active morphology, with a thickening of cell body and shortening and thickening of microglial processes (Figure 3A). Furthermore, the increased expression of *Clec7a*, *Cd45*, *Trem2* and *Lgals3* corroborated microglial activation after 1-month treatment (Figure 3B). We should take into account that, unlike previously mentioned publications, we maintained the depletion inductor, so a continuous microglial depletion and repopulation was taking place. An active phenotype is typical of phagocytic microglia, which could be eliminating cellular debris of dead microglia [32]. In this sense, an upregulation of scavenger-associated proteins, such as Cd36 [68], and of the phagocytic marker Cd68 [49] have been reported in repopulating microglia. However, given the small remaining microglial population at some points (for instance, at the end of our acute treatment (Figure 1A,B)), the CNS must count with an additional mechanism to eliminate all cellular debris, among which phagocytic activity of astrocytes has been proposed [69,89]. Konishi et al. (2020) demonstrated that, after specific microglial depletion (using Siglech^dtr^ mice), astrocytes, rather than CNS-associated macrophages or circulating monocytes, clear microglial debris [69].

Finally, when drawing conclusion from chronic depletion models, we must be aware that changes observed are difficult to be unequivocally addressed to one factor. They may be due to: (i) a reduction in total microglial levels, (ii) a reduction of previous, sometimes burnt out, microglia, or (iii) the presence of emerging microglia whose characteristics are still not thoroughly described. Same challenges will occur with other state-of-the-art microglial manipulation techniques, such as chimeric mice. These models have been proposed as emerging tools to substitute exhausted microglia and/or to characterize human microglial response in AD pathology. Under this approach, mouse microglia is depleted to be replaced with human iPS-derived microglia in immunosuppressed mice [90]. After this manipulation, alterations in the progression of AD pathologies could be addressed to: (i) a reduction in the number of mouse microglia, (ii) the effect of human microglia, (iii) the immunosuppression of mice, (iv) the interaction between mouse and human microglia. Additionally, taking into account the stablished interaction between microglia and astrocytes [91], in depletion as well as in chimeric mice, modifications in the pathologies may also be caused by changes occurred in other glial cells. In essence, although they are promising tools, we should be cautious when drawing conclusions from these models. 

## 6. Do Microglia Refresh or Poison AD Progression?

In order to further characterize microglial role in AD, microglial depletion models are being combined with AD mouse models bearing either Aβ and/or Tau pathologies (Table 1). Currently, controversial data are found and new studies are needed to clarify if microglia is beneficial or detrimental to AD pathology.

**Table 1:** Relevant results in AD mouse models of microglia depletion. Mouse models used and main outcomes are shown.

Pathology	Depletion Model	Outcomes	References
Aβ	PLX5622 in 5xFAD mice, from 4- to 5-month-old.	50% microglia depletion. Reduction of microgliosis and plaque burden, enhancement of neuritic dystrophies.	[62]
Aβ	PLX3397 in 5xFAD mice, from 9- to 10-month-old.	Around 50% microglia depletion. Decrease in Aβ deposition and rescue of dopaminergic signaling.	[65]
Aβ	PLX5622 in APP/PS1 mice, from 12- to 13-month-old.	Diminution of leukotriene biosynthesis and the neuronal 5-lipoxygenase.	[92]
Aβ	PLX5622 in 5xFAD mice from 1.5 to 4- or 7-month-olds.	97% microglia depletion. Reduction of plaque deposition, but increase of cerebral amyloid angiopathy formation.	[45]
Aβ	PLX3397 in 5xFAD mice from 2- to 5-month-old.	70–80% microglia depletion. Reduction of intraneuronal amyloid, neuritic plaque deposition and improvement in cognitive functions (fear conditioning tests).	[93]
Aβ	Diphtheria toxin in 15 months-old *Cx3cr1^CreER/+^:R26^DTR/+^*/APPxPS1 mice, for 1–2 weeks.	90% depletion. No changes in the number of Aβ plaques, but an increase in size.	[94]
Aβ	GWS2580 in APP/PS1 mice from 6- to 9-month-old.	30% reduction of microglia. No changes in the number of Aβ plaques. Improved performance in memory and behavioral tasks.	[43]
Aβ	PLX3397 in 5xFAD from 10- to 11-month-old.	90% microglia depletion. No alterations in β-amyloid levels or plaque load, but rescue of dendritic spine loss and improvements in contextual memory.	[56]
Aβ and Tau	PLX5562 in 3xTg mice for 3 months.	30% microglia depletion. No changes in total or phosphorylated Tau. Improvements in cognition.	[64]
Aβ and Tau	PLX3397 from 5.5- to 7-month-old in 5xFAD/PS19 Tau -injected mice.	81% microglia depletion. Higher reduction in non-plaque-associated microglia. No changes in Aβ pathology, reduction in Tau pathology and neurodegeneration.	[71]
Aβ and Tau	PLX3397 from 6- to 9-month-old in 5xFAD mice injected with AD-Tau.	Improved cognitive and neuronal deficits. Enhancement of Tau seeding and spreading around plaques.	[88]
Tau	*Cx3cr1CreER/R26DTA*/*hTAU* mice, treated with tamoxifen for 2–3 months at different ages.	60% microglia depletion. No changes in soluble oligomeric, phosphorylated or total aggregated Tau levels.	[66]
Tau	PLX3397 in P301S *APOE E4* mice from 6- to 9-month-old.	Total microglia depletion. Protection from brain volume loss and neurodegeneration. Reduction of Tau pathology progression.	[49]
Tau	PLX3397 in rTg4510 mice, from 12- to 15-month-old.	30% microglia depletion. No changes in Tau burden, cortical atrophy, blood vessels or glial activation.	[63]
Tau	(a) Clodronate liposomes and PLX3397 in AAV-GFP/Tau injected C57BL/6 mice. (b) PLX3397 in PS19 mice. In both cases, from 3.5- to 4.5-month-old.	70–80% (a) and 90% (b) microglia depletion. Reduction of phospho-Tau.	[95]

### 6.1. Microglial Interplay with Aβ Pathology Progression

Recently, it has been shown that immune cells located around Aβ plaques are exclusively microglia, without any contribution of infiltrating myeloid cells [96,97], so then microglia would be essential in the containment of amyloid deposits and could have a protective role. Huang et al. (2021) have demonstrated that microglia, through TAM receptor tyrosine kinases such as Axl and Mer, engulf amyloid plaques and promote dense-core plaque development [98]. In this line, a diminution of Aβ plaques has been reported when CSF1R inhibitors are administered before or at the beginning of the pathology [45,62]. These authors showed that total Aβ charge did not vary, but there were changes in its distribution, which supports the importance of microglia in the formation of Aβ plaques. Actually, PLX3997 administration to 2-months-old 5xFAD mice for 3 months inhibited plaque formation [93] and, more importantly, microglia repopulation in 5–6 months-old 5xFAD mice reverted plaque morphologies to control levels [62]. Additionally, microglia may intervene by secreting aggregation factors that help with Aβ clearance and by internalizing low concentrations of fibrillary Aβ, potentially more toxic [99,100]. However, most of publications based on microglial depletion in Aβ mouse models revealed no changes in total Aβ burden (Table 1), indicating that microglia might not be the principal source of β-amyloid clearance from the CNS [56]. 

If these changes in Aβ distribution directly affect neuronal pathology is still a matter of debate. Casali et al. (2020) have reported that microglial depletion in 5xFAD animals translated into an increase of neuritic dystrophies [62], while other studies observed a decrease in intraneuronal Aβ and neuritic plaques, accompanied by an amelioration of cognitive decline [93]. In the same line, an amelioration of spinal and neuronal loss [88] and an improvement in memory tasks have been reported following microglia depletion [43,56]. Distinct microglia populations may be mediating diverse effects in Aβ and neuronal pathologies progression. While plaque-associated microglia may be protective, non-plaque-associated microglia, also in the presence of Tau pathology, could display neurotoxic functions. In this sense, it has been recently demonstrated that the preferential depletion of microglia distal to amyloid deposits significantly attenuated Tau pathology and neuronal atrophy in Tau-seeding 5xFAD/PS19 mice [71]. In this model, while Aβ-associated DAM microglia may compact amyloid plaques and limit its toxicity, non-plaque-associated microglia may contribute to Tau spread and to a pro-inflammatory environment that could mediate neuronal dysfunction. Furthermore, microglia surrounding amyloid deposits may finally become exhausted due to local plaque-associated hypoxia [18] and, then, also induce neurotoxicity. Therefore, some of the contradictory findings above described are not surprising given the heterogeneity of microglial subsets described in the context of AD.

Once Aβ pathology is established (mice from 10- to 18 month-old), some works showed an improvement in cognitive abilities when microglial population was reduced, although it was not accompanied by a decrease in amyloid load [56]. In contrast, Zhao et al. (2017) showed that, in spite of no changes in the number of Aβ plaques, there was an increase in their size [94], once again giving microglia a fundamental role in Aβ plaques compaction. 

In short, despite further work is required, most of studies did not reveal significant changes in total Aβ load after microglial depletion. However, an improvement in neuronal pathology has been frequently reported, which give us hope about the efficacy of microglia replacement in AD therapy. It would be of great interest to determine specific markers which allow the precise identification of highly neurotoxic and/or degenerated plaque-associated microglia, in order to specifically deplete them. Microglial proliferation which repopulate the depleted niche rapidly acquire mature characteristics [58,101] and may counteract AD pathology by better containing amyloid deposits and diminishing pathological phagocytosis of synapses, then contributing to the amelioration of AD cognitive decline. 

### 6.2. Microglial Contribution to Tau Pathology

In spite of being Tau pathology the one that best correlates with AD cognitive decline, the role of microglia in the progression of Tau deposits is far from being elucidated. A direct interaction between microglia and Tau forms has been widely proposed, but neither the underlying mechanisms nor the specific Tau species of interaction are clearly described. It has been revised how microglia could phagocytose extracellular Tau by the CX3CR1 receptor, as the aminoacidic sequence of Tau display a 37% homology with the CX3CL1 sequence [102]. However, this will be hampered at late AD stages due to Tau hyperphosphorylation and CX3CL1 overexpression [103]. On the other hand, Nagamine et al. (2016) suggested an interaction between neurofibrillary tangles and microglial cells through CD33 receptor, which is capable of recognizing the sialic acid residues that appear in the neurofibrillary tangles [104]. Additionally, more and more evidences suggest a contribution of microglia in the spreading of Tau seeds [95,105,106]. In contrast, Tau antibodies boost microglial phagocytosis of Tau in vitro [107,108] and passive immunization reduced Tau deposits in an AD model [109].

Due to controversial data, it is of great interest to evaluate the influence of microglial depletion and repopulation on the onset and progression of Tau pathology (see Table 1). Zhu et al. (2020) have not observed variations in Tau pathology after microglial depletion or engraftment of peripherally-derived macrophages [66], but the *hTAU* model used is less aggressive than most transgenic murine models which express the mutant Tau form associated with frontotemporal dementia: JNPL3 [110,111], PS19 [112] or rTg4510 [113]. In fact, in the *hTAU* model, a homeostatic microglial phenotype was described even at later ages [66]. On the other hand, no changes were observed in Tau pathology in the rTg4510 [63] or 3xTg-AD [64] models after a 30% microglial depletion using the pharmacological inhibitors PLX3397 and PLX5562, respectively. 

Although deficiencies in specific microglial genes involved in their survival and proliferation, such as *Cx3cr1* or *Trem2*, worsened Tau pathology [114,115,116], microglial depletion in PS19 mice coursed with an amelioration of Tau pathology [95]. In the same line, Shi et al. (2019) have reported an amelioration of Tau pathology and brain volume loss in P301S *APOE E4* mice after 3 months of PLX3397 treatment [49]. In contrast, Gratuze et al. (2021) have recently showed that microglia depletion and even repopulating microglia enhance Tau seeding and spreading around plaques in a Tau-injected amyloidogenic model [88]. This discrepancy may suggest that amyloid pathology modifies the role of microglia in Tau spreading. Then, further experiments are required to stablish if microglia-mediated inflammation may influence Tau progression and subsequent neurodegeneration. 

Studies of microglial depletion in other CNS pathologies showed similarly controversial results. Rice et al. (2017) and Acharya et al. (2016) described significant improvements subsequent to microglial depletion in neuronal damage models [59,117]. In the same line, Li et al. (2017) showed neuroprotection following microglial depletion in a model of intracerebral hemorrhage [118]. However, other publications reported an increase in neuroinflammation and brain damage after microglial depletion in ischemia models [119,120].

### 6.3. Is Microglia Renewal a Promising Therapeutic Approach for AD?

If the microglial depletion–repopulation phenomenon was able to slow down the progression of Aβ and Tau pathologies, therapies based on microglial turnover should be designed. In both, microglia hyperactivation or dysfunction, microglial replacement would improve CNS homeostasis. Recently, different strategies for microglial replacement in murine models were reviewed [90]. In humans, CSF1R inhibitor PLX3397 has shown some efficacy in microglial depletion and has been approved by the FDA for recurrent glioblastoma treatment [121]. Although microglia proliferation is quite limited in the adult human brain, it has been estimated that a 28% of microglia is renowned daily, being the lifespan of these cells 4.2 years [6]. Actually, Olmos-Alonso et al. (2016) have reported a significant increment in microglia proliferation in AD samples compared to controls, being the proliferation rate 2.63% in grey matter in AD brains [43]. This increase could be trying to compensate microglial degeneration at late Braak stages [28,122]. In addition, the Trem2 agonist AL002, which enhances microglial proliferation in murine models [123], is being tested in phase I to treat mild and moderate AD stages (https://clinicaltrials.gov/ct2/show/NCT03635047, accessed on 5 July 2021), so it would be a candidate to favor the repopulation of the depleted niche, thus promoting the scarce microglial proliferation that takes place in humans. 

Furthermore, microglial activation has been proposed as responsible for Aβ clearance under anti-Aβ immunotherapy treatments in AD. In fact, Aducanumab, recently approved by the FDA, increases microglial number around Aβ plaques in Tg2576 mice, suggesting phagocytosis of antibody-Aβ complexes as a clearance mechanism [124]. This microglial recruitment has also been described for Gantenerumab, another anti-Aβ immunotherapy in clinical phase III [125,126,127].

Therefore, novel approaches to increase microglial renewal may be a promising tool to assess microglial role in the ATN continuum and to design potential therapies for AD. 

## 7. Concluding Remarks

Different approaches to deplete microglia in the mouse brain are based on pharmacological or genetic manipulation methods. Although generating new transgenic models takes longer, cell-targeted genetic strategies are more specific and avoid mainly undesired effects in other cell types or tissues. So far, diverse strategies have given rise to a variable percentage of depletion, which should also be taken into account before selecting a depletion model. Our research group has generated *Cx3cr1^CreER^/Csf1r^flx/flx^* mice, which provided temporal control of microglia depletion by using tamoxifen, either by intraperitoneal injection (acute depletion) or by chronic oral administration. Our results reported an important microglial repopulation, so remaining and emerging microglia constantly coexist in chronic models. Although we cannot totally reject a peripheral origin, the most accepted hypothesis is that a rapid microglial self-renewal is occurring. Further studies are required to exactly determine the mechanisms underlying microglial recolonization and to explain the intriguing repopulation pattern that we observed. 

A sustained microglial depletion would be essential to study a context of microglial dysfunction in AD and other chronic neurodegenerative diseases. However, due to the high microglial proliferation capacity observed, depletion models may better represent a context of microglial renewal. Up to date, controversial data from AD microglial depletion models have hindered the specific contribution of microglia on the ATN continuum. However, if we were able to slow down Aβ and Tau progression by enhancing microglial turnover, microglial depletion and repopulation strategies would be a promising therapeutic strategy for AD.

## Supplementary Materials

The following are available online at https://www.mdpi.com/article/10.3390/ijms22189734/s1.

## References

[1] Nguyen A.T., Wang K., Hu G., Wang X., Miao Z., Azevedo J.A., Suh E., Van Deerlin V.M., Choi D., Roeder K. (2020). APOE and TREM2 Regulate Amyloid-Responsive Microglia in Alzheimer’s Disease. Acta Neuropathol..

[2] Wang H., Dey K.K., Chen P.-C., Li Y., Niu M., Cho J.-H., Wang X., Bai B., Jiao Y., Chepyala S.R. (2020). Integrated Analysis of Ultra-Deep Proteomes in Cortex, Cerebrospinal Fluid and Serum Reveals a Mitochondrial Signature in Alzheimer’s Disease. Mol. Neurodegener..

[3] Ginhoux F., Greter M., Leboeuf M., Nandi S., See P., Gokhan S., Mehler M.F., Conway S.J., Ng L.G., Stanley E.R. (2010). Fate Mapping Analysis Reveals That Adult Microglia Derive from Primitive Macrophages. Science.

[4] Lawson L.J., Perry V.H., Dri P., Gordon S. (1990). Heterogeneity in the Distribution and Morphology of Microglia in the Normal Adult Mouse Brain. Neuroscience.

[5] Askew K., Li K., Olmos-Alonso A., Garcia-Moreno F., Liang Y., Richardson P., Tipton T., Chapman M.A., Riecken K., Beccari S. (2017). Coupled Proliferation and Apoptosis Maintain the Rapid Turnover of Microglia in the Adult Brain. Cell Rep..

[6] Réu P., Khosravi A., Bernard S., Mold J.E., Salehpour M., Alkass K., Perl S., Tisdale J., Possnert G., Druid H. (2017). The Lifespan and Turnover of Microglia in the Human Brain. Cell Rep..

[7] Jack C.R., Knopman D.S., Jagust W.J., Shaw L.M., Aisen P.S., Weiner M.W., Petersen R.C., Trojanowski J.Q. (2010). Hypothetical Model of Dynamic Biomarkers of the Alzheimer’s Pathological Cascade. Lancet Neurol..

[8] He Z., Guo J.L., McBride J.D., Narasimhan S., Kim H., Changolkar L., Zhang B., Gathagan R.J., Yue C., Dengler C. (2018). Amyloid-β Plaques Enhance Alzheimer’s Brain Tau-Seeded Pathologies by Facilitating Neuritic Plaque Tau Aggregation. Nat. Med..

[9] Clayton K., Delpech J.C., Herron S., Iwahara N., Ericsson M., Saito T., Saido T.C., Ikezu S., Ikezu T. (2021). Plaque Associated Microglia Hyper-Secrete Extracellular Vesicles and Accelerate Tau Propagation in a Humanized APP Mouse Model. Mol. Neurodegener..

[10] Mathys H., Davila-Velderrain J., Peng Z., Gao F., Mohammadi S., Young J.Z., Menon M., He L., Abdurrob F., Jiang X. (2019). Single-Cell Transcriptomic Analysis of Alzheimer’s Disease. Nature.

[11] Grubman A., Chew G., Ouyang J.F., Sun G., Choo X.Y., McLean C., Simmons R.K., Buckberry S., Vargas-Landin D.B., Poppe D. (2019). A Single-Cell Atlas of Entorhinal Cortex from Individuals with Alzheimer’s Disease Reveals Cell-Type-Specific Gene Expression Regulation. Nat. Neurosci..

[12] Masuda T., Sankowski R., Staszewski O., Böttcher C., Amann L., Sagar, Scheiwe C., Nessler S., Kunz P., van Loo G. (2019). Spatial and Temporal Heterogeneity of Mouse and Human Microglia at Single-Cell Resolution. Nature.

[13] Leng F., Edison P. (2021). Neuroinflammation and Microglial Activation in Alzheimer Disease: Where Do We Go from Here?. Nat. Rev. Neurol..

[14] Zhou Y., Song W.M., Andhey P.S., Swain A., Levy T., Miller K.R., Poliani P.L., Cominelli M., Grover S., Gilfillan S. (2020). Human and Mouse Single-Nucleus Transcriptomics Reveal TREM2-Dependent and—Independent Cellular Responses in Alzheimer’s Disease. Nat. Med..

[15] Keren-Shaul H., Spinrad A., Weiner A., Matcovitch-Natan O., Dvir-Szternfeld R., Ulland T.K., David E., Baruch K., Lara-Astaiso D., Toth B. (2017). A Unique Microglia Type Associated with Restricting Development of Alzheimer’s Disease. Cell.

[16] Condello C., Yuan P., Schain A., Grutzendler J. (2015). Microglia Constitute a Barrier That Prevents Neurotoxic Protofibrillar Aβ42 Hotspots around Plaques. Nat. Commun..

[17] Deczkowska A., Keren-Shaul H., Weiner A., Colonna M., Schwartz M., Amit I. (2018). Disease-Associated Microglia: A Universal Immune Sensor of Neurodegeneration. Cell.

[18] March-Diaz R., Lara-Ureña N., Romero-Molina C., Heras-Garvin A., Luis C.O.S., Alvarez-Vergara M.I., Sanchez-Garcia M.A., Sanchez-Mejias E., Davila J.C., Rosales-Nieves A.E. (2021). Hypoxia Compromises the Mitochondrial Metabolism of Alzheimer’s Disease Microglia via HIF1. Nat. Aging.

[19] Friedman B.A., Srinivasan K., Ayalon G., Meilandt W.J., Lin H., Huntley M.A., Cao Y., Lee S.-H., Haddick P.C.G., Ngu H. (2018). Diverse Brain Myeloid Expression Profiles Reveal Distinct Microglial Activation States and Aspects of Alzheimer’s Disease Not Evident in Mouse Models. Cell Rep..

[20] Romero-Molina C., Navarro V., Sanchez-Varo R., Jimenez S., Fernandez-Valenzuela J.J., Sanchez-Mico M.V., Muñoz-Castro C., Gutierrez A., Vitorica J., Vizuete M. (2018). Distinct Microglial Responses in Two Transgenic Murine Models of TAU Pathology. Front. Cell. Neurosci..

[21] Leyns C.E.G., Gratuze M., Narasimhan S., Jain N., Koscal L.J., Jiang H., Manis M., Colonna M., Lee V.M.Y., Ulrich J.D. (2019). TREM2 Function Impedes Tau Seeding in Neuritic Plaques. Nat. Neurosci..

[22] Serrano-Pozo A., Gómez-Isla T., Growdon J.H., Frosch M.P., Hyman B.T. (2013). A Phenotypic Change but Not Proliferation Underlies Glial Responses in Alzheimer Disease. Am. J. Pathol..

[23] Hamelin L., Lagarde J., Dorothée G., Leroy C., Labit M., Comley R.A., de Souza L.C., Corne H., Dauphinot L., Bertoux M. (2016). Early and Protective Microglial Activation in Alzheimer’s Disease: A Prospective Study Using 18F-DPA-714 PET Imaging. Brain J. Neurol..

[24] Fan Z., Brooks D.J., Okello A., Edison P. (2017). An Early and Late Peak in Microglial Activation in Alzheimer’s Disease Trajectory. Brain.

[25] Navarro V., Sanchez-Mejias E., Jimenez S., Muñoz-Castro C., Sanchez-Varo R., Davila J.C., Vizuete M., Gutierrez A., Vitorica J. (2018). Microglia in Alzheimer’s Disease: Activated, Dysfunctional or Degenerative. Front. Aging Neurosci..

[26] Jimenez S., Baglietto-Vargas D., Caballero C., Moreno-Gonzalez I., Torres M., Sanchez-Varo R., Ruano D., Vizuete M., Gutierrez A., Vitorica J. (2008). Inflammatory Response in the Hippocampus of PS1M146L/APP751SL Mouse Model of Alzheimer’s Disease: Age-Dependent Switch in the Microglial Phenotype from Alternative to Classic. J. Neurosci. Off. J. Soc. Neurosci..

[27] Galatro T.F., Holtman I.R., Lerario A.M., Vainchtein I.D., Brouwer N., Sola P.R., Veras M.M., Pereira T.F., Leite R.E.P., Möller T. (2017). Transcriptomic Analysis of Purified Human Cortical Microglia Reveals Age-Associated Changes. Nat. Neurosci..

[28] Sanchez-Mejias E., Navarro V., Jimenez S., Sanchez-Mico M., Sanchez-Varo R., Nuñez-Diaz C., Trujillo-Estrada L., Davila J.C., Vizuete M., Gutierrez A. (2016). Soluble Phospho-Tau from Alzheimer’s Disease Hippocampus Drives Microglial Degeneration. Acta Neuropathol..

[29] Sierksma A., Escott-Price V., Strooper B.D. (2020). Translating Genetic Risk of Alzheimer’s Disease into Mechanistic Insight and Drug Targets. Science.

[30] Gutierrez A., Vitorica J. (2018). Toward a New Concept of Alzheimer’s Disease Models: A Perspective from Neuroinflammation. J. Alzheimers Dis..

[31] Bohlen C.J., Bennett F.C., Tucker A.F., Collins H.Y., Mulinyawe S.B., Barres B.A. (2017). Diverse Requirements for Microglial Survival, Specification, and Function Revealed by Defined-Medium Cultures. Neuron.

[32] Elmore M.R.P., Najafi A.R., Koike M.A., Dagher N.N., Spangenberg E.E., Rice R.A., Kitazawa M., Matusow B., Nguyen H., West B.L. (2014). CSF1 Receptor Signaling Is Necessary for Microglia Viability, Which Unmasks a Cell That Rapidly Repopulates the Microglia-Depleted Adult Brain. Neuron.

[33] Wu L., Li Y., Yu M., Yang F., Tu M., Xu H. (2018). Notch Signaling Regulates Microglial Activation and Inflammatory Reactions in a Rat Model of Temporal Lobe Epilepsy. Neurochem. Res..

[34] Elegheert J., Desfosses A., Shkumatov A.V., Wu X., Bracke N., Verstraete K., Van Craenenbroeck K., Brooks B.R., Svergun D.I., Vergauwen B. (2011). Extracellular Complexes of the Hematopoietic Human and Mouse CSF-1 Receptor Are Driven by Common Assembly Principles. Structure.

[35] Felix J., De Munck S., Verstraete K., Meuris L., Callewaert N., Elegheert J., Savvides S.N. (2015). Structure and Assembly Mechanism of the Signaling Complex Mediated by Human CSF-1. Structure.

[36] Ma X., Lin W.Y., Chen Y., Stawicki S., Mukhyala K., Wu Y., Martin F., Bazan J.F., Starovasnik M.A. (2012). Structural Basis for the Dual Recognition of Helical Cytokines IL-34 and CSF-1 by CSF-1R. Structure.

[37] Patel S., Player M. (2009). Colony-Stimulating Factor-1 Receptor Inhibitors for the Treatment of Cancer and Inflammatory Disease. Curr. Top. Med. Chem..

[38] Chitu V., Stanley E.R., Jenny A. (2017). Chapter Seven—Regulation of Embryonic and Postnatal Development by the CSF-1 Receptor. Current Topics in Developmental Biology.

[39] Erblich B., Zhu L., Etgen A.M., Dobrenis K., Pollard J.W. (2011). Absence of Colony Stimulation Factor-1 Receptor Results in Loss of Microglia, Disrupted Brain Development and Olfactory Deficits. PLoS ONE.

[40] Green K.N., Crapser J.D., Hohsfield L.A. (2020). To Kill a Microglia: A Case for CSF1R Inhibitors. Trends Immunol..

[41] Wu W., Li Y., Wei Y., Bosco D.B., Xie M., Zhao M.-G., Richardson J.R., Wu L.-J. (2020). Microglial Depletion Aggravates the Severity of Acute and Chronic Seizures in Mice. Brain. Behav. Immun..

[42] Han J., Zhu K., Zhang X.-M., Harris R.A. (2019). Enforced Microglial Depletion and Repopulation as a Promising Strategy for the Treatment of Neurological Disorders. Glia.

[43] Olmos-Alonso A., Schetters S.T.T., Sri S., Askew K., Mancuso R., Vargas-Caballero M., Holscher C., Perry V.H., Gomez-Nicola D. (2016). Pharmacological Targeting of CSF1R Inhibits Microglial Proliferation and Prevents the Progression of Alzheimer’s-like Pathology. Brain J. Neurol..

[44] Mancuso R., Fryatt G., Cleal M., Obst J., Pipi E., Monzón-Sandoval J., Ribe E., Winchester L., Webber C., Nevado A. (2019). CSF1R Inhibitor JNJ-40346527 Attenuates Microglial Proliferation and Neurodegeneration in P301S Mice. Brain.

[45] Spangenberg E., Severson P.L., Hohsfield L.A., Crapser J., Zhang J., Burton E.A., Zhang Y., Spevak W., Lin J., Phan N.Y. (2019). Sustained Microglial Depletion with CSF1R Inhibitor Impairs Parenchymal Plaque Development in an Alzheimer’s Disease Model. Nat. Commun..

[46] Lei F., Cui N., Zhou C., Chodosh J., Vavvas D.G., Paschalis E.I. (2020). CSF1R Inhibition by a Small-Molecule Inhibitor Is Not Microglia Specific; Affecting Hematopoiesis and the Function of Macrophages. Proc. Natl. Acad. Sci. USA.

[47] Thompson M.L., Jimenez-Andrade J.M., Chartier S., Tsai J., Burton E.A., Habets G., Lin P.S., West B.L., Mantyh P.W. (2015). Targeting Cells of the Myeloid Lineage Attenuates Pain and Disease Progression in a Prostate Model of Bone Cancer. Pain.

[48] Chitu V., Gokhan Ş., Nandi S., Mehler M.F., Stanley E.R. (2016). Emerging Roles for CSF-1 Receptor and Its Ligands in the Nervous System. Trends Neurosci..

[49] Shi Y., Manis M., Long J., Wang K., Sullivan P.M., Serrano J.R., Hoyle R., Holtzman D.M. (2019). Microglia Drive APOE-Dependent Neurodegeneration in a Tauopathy Mouse Model. J. Exp. Med..

[50] Jäkel S., Dimou L. (2017). Glial Cells and Their Function in the Adult Brain: A Journey through the History of Their Ablation. Front. Cell. Neurosci..

[51] Gowing G., Vallières L., Julien J.-P. (2006). Mouse Model for Ablation of Proliferating Microgliain Acute CNS Injuries. Glia.

[52] Bruttger J., Karram K., Wörtge S., Regen T., Marini F., Hoppmann N., Klein M., Blank T., Yona S., Wolf Y. (2015). Genetic Cell Ablation Reveals Clusters of Local Self-Renewing Microglia in the Mammalian Central Nervous System. Immunity.

[53] Kim H., Kim M., Im S.-K., Fang S. (2018). Mouse Cre-LoxP System: General Principles to Determine Tissue-Specific Roles of Target Genes. Lab. Anim. Res..

[54] Cronk J.C., Filiano A.J., Louveau A., Marin I., Marsh R., Ji E., Goldman D.H., Smirnov I., Geraci N., Acton S. (2018). Peripherally Derived Macrophages Can Engraft the Brain Independent of Irradiation and Maintain an Identity Distinct from Microglia. J. Exp. Med..

[55] Kaiser T., Feng G. (2019). Tmem119-EGFP and Tmem119-CreERT2 Transgenic Mice for Labeling and Manipulating Microglia. eNeuro.

[56] Spangenberg E.E., Lee R.J., Najafi A.R., Rice R.A., Elmore M.R.P., Blurton-Jones M., West B.L., Green K.N. (2016). Eliminating Microglia in Alzheimer’s Mice Prevents Neuronal Loss without Modulating Amyloid-β Pathology. Brain J. Neurol..

[57] Yao Y., Echeverry S., Shi X.Q., Yang M., Yang Q.Z., Wang G.Y.F., Chambon J., Wu Y.C., Fu K.Y., De Koninck Y. (2016). Dynamics of Spinal Microglia Repopulation Following an Acute Depletion. Sci. Rep..

[58] Elmore M.R.P., Lee R.J., West B.L., Green K.N. (2015). Characterizing Newly Repopulated Microglia in the Adult Mouse: Impacts on Animal Behavior, Cell Morphology, and Neuroinflammation. PLoS ONE.

[59] Rice R.A., Pham J., Lee R.J., Najafi A.R., West B.L., Green K.N. (2017). Microglial Repopulation Resolves Inflammation and Promotes Brain Recovery after Injury. Glia.

[60] Grathwohl S.A., Kälin R.E., Bolmont T., Prokop S., Winkelmann G., Kaeser S.A., Odenthal J., Radde R., Eldh T., Gandy S. (2009). Formation and Maintenance of Alzheimer’s Disease Beta-Amyloid Plaques in the Absence of Microglia. Nat. Neurosci..

[61] Faustino J.V., Wang X., Johnson C.E., Klibanov A., Derugin N., Wendland M.F., Vexler Z.S. (2011). Microglial Cells Contribute to Endogenous Brain Defenses after Acute Neonatal Focal Stroke. J. Neurosci. Off. J. Soc. Neurosci..

[62] Casali B.T., MacPherson K.P., Reed-Geaghan E.G., Landreth G.E. (2020). Microglia Depletion Rapidly and Reversibly Alters Amyloid Pathology by Modification of Plaque Compaction and Morphologies. Neurobiol. Dis..

[63] Bennett R.E., Bryant A., Hu M., Robbins A.B., Hopp S.C., Hyman B.T. (2018). Partial Reduction of Microglia Does Not Affect Tau Pathology in Aged Mice. J. Neuroinflamm..

[64] Dagher N.N., Najafi A.R., Kayala K.M.N., Elmore M.R.P., White T.E., Medeiros R., West B.L., Green K.N. (2015). Colony-Stimulating Factor 1 Receptor Inhibition Prevents Microglial Plaque Association and Improves Cognition in 3xTg-AD Mice. J. Neuroinflamm..

[65] Son Y., Jeong Y.J., Shin N.-R., Oh S.J., Nam K.R., Choi H.-D., Choi J.Y., Lee H.-J. (2020). Inhibition of Colony-Stimulating Factor 1 Receptor by PLX3397 Prevents Amyloid Beta Pathology and Rescues Dopaminergic Signaling in Aging 5xFAD Mice. Int. J. Mol. Sci..

[66] Zhu K., Pieber M., Han J., Blomgren K., Zhang X.-M., Harris R.A., Lund H. (2020). Absence of Microglia or Presence of Peripherally-Derived Macrophages Does Not Affect Tau Pathology in Young or Old HTau Mice. Glia.

[67] Najafi A.R., Crapser J., Jiang S., Ng W., Mortazavi A., West B.L., Green K.N. (2018). A Limited Capacity for Microglial Repopulation in the Adult Brain. Glia.

[68] Huang Y., Xu Z., Xiong S., Sun F., Qin G., Hu G., Wang J., Zhao L., Liang Y.-X., Wu T. (2018). Repopulated Microglia Are Solely Derived from the Proliferation of Residual Microglia after Acute Depletion. Nat. Neurosci..

[69] Konishi H., Okamoto T., Hara Y., Komine O., Tamada H., Maeda M., Osako F., Kobayashi M., Nishiyama A., Kataoka Y. (2020). Astrocytic Phagocytosis Is a Compensatory Mechanism for Microglial Dysfunction. EMBO J..

[70] Zhan L., Krabbe G., Du F., Jones I., Reichert M.C., Telpoukhovskaia M., Kodama L., Wang C., Cho S., Sayed F. (2019). Proximal Recolonization by Self-Renewing Microglia Re-Establishes Microglial Homeostasis in the Adult Mouse Brain. PLoS Biol..

[71] Lodder C., Scheyltjens I., Stancu I.C., Botella Lucena P., Gutiérrez de Ravé M., Vanherle S., Vanmierlo T., Cremers N., Vanrusselt H., Brône B. (2021). CSF1R Inhibition Rescues Tau Pathology and Neurodegeneration in an A/T/N Model with Combined AD Pathologies, While Preserving Plaque Associated Microglia. Acta Neuropathol. Commun..

[72] Wang Y., Cella M., Mallinson K., Ulrich J.D., Young K.L., Robinette M.L., Gilfillan S., Krishnan G.M., Sudhakar S., Zinselmeyer B.H. (2015). TREM2 Lipid Sensing Sustains the Microglial Response in an Alzheimer’s Disease Model. Cell.

[73] Zhan L., Fan L., Kodama L., Sohn P.D., Wong M.Y., Mousa G.A., Zhou Y., Li Y., Gan L. (2020). A MAC2-Positive Progenitor-like Microglial Population Is Resistant to CSF1R Inhibition in Adult Mouse Brain. eLife.

[74] Shemer A., Grozovski J., Tay T.L., Tao J., Volaski A., Süß P., Ardura-Fabregat A., Gross-Vered M., Kim J.-S., David E. (2018). Engrafted Parenchymal Brain Macrophages Differ from Microglia in Transcriptome, Chromatin Landscape and Response to Challenge. Nat. Commun..

[75] Gosselin D., Link V.M., Romanoski C.E., Fonseca G.J., Eichenfield D.Z., Spann N.J., Stender J.D., Chun H.B., Garner H., Geissmann F. (2014). Environment Drives Selection and Function of Enhancers Controlling Tissue-Specific Macrophage Identities. Cell.

[76] Varvel N.H., Grathwohl S.A., Baumann F., Liebig C., Bosch A., Brawek B., Thal D.R., Charo I.F., Heppner F.L., Aguzzi A. (2012). Microglial Repopulation Model Reveals a Robust Homeostatic Process for Replacing CNS Myeloid Cells. Proc. Natl. Acad. Sci. USA.

[77] Lund H., Pieber M., Parsa R., Han J., Grommisch D., Ewing E., Kular L., Needhamsen M., Espinosa A., Nilsson E. (2018). Competitive Repopulation of an Empty Microglial Niche Yields Functionally Distinct Subsets of Microglia-like Cells. Nat. Commun..

[78] Plemel J.R., Stratton J.A., Michaels N.J., Rawji K.S., Zhang E., Sinha S., Baaklini C.S., Dong Y., Ho M., Thorburn K. (2020). Microglia Response Following Acute Demyelination Is Heterogeneous and Limits Infiltrating Macrophage Dispersion. Sci. Adv..

[79] Rajan W.D., Wojtas B., Gielniewski B., Miró-Mur F., Pedragosa J., Zawadzka M., Pilanc P., Planas A.M., Kaminska B. (2020). Defining Molecular Identity and Fates of CNS-Border Associated Macrophages after Ischemic Stroke in Rodents and Humans. Neurobiol. Dis..

[80] Miyazaki T., Ishikawa E., Matsuda M., Sugii N., Kohzuki H., Akutsu H., Sakamoto N., Takano S., Matsumura A. (2020). Infiltration of CD163-Positive Macrophages in Glioma Tissues after Treatment with Anti-PD-L1 Antibody and Role of PI3Kγ Inhibitor as a Combination Therapy with Anti-PD-L1 Antibody in in Vivo Model Using Temozolomide-Resistant Murine Glioma-Initiating Cells. Brain Tumor Pathol..

[81] Swanson M.E.V., Scotter E.L., Smyth L.C.D., Murray H.C., Ryan B., Turner C., Faull R.L.M., Dragunow M., Curtis M.A. (2020). Identification of a Dysfunctional Microglial Population in Human Alzheimer’s Disease Cortex Using Novel Single-Cell Histology Image Analysis. Acta Neuropathol. Commun..

[82] Tan Y.-L., Yuan Y., Tian L. (2020). Microglial Regional Heterogeneity and Its Role in the Brain. Mol. Psychiatry.

[83] Thion M.S., Ginhoux F., Garel S. (2018). Microglia and Early Brain Development: An Intimate Journey. Science.

[84] Hu Y., Fryatt G.L., Ghorbani M., Obst J., Menassa D.A., Martin-Estebane M., Muntslag T.A.O., Olmos-Alonso A., Guerrero-Carrasco M., Thomas D. (2021). Replicative Senescence Dictates the Emergence of Disease-Associated Microglia and Contributes to Aβ Pathology. Cell Rep..

[85] Ghosh S., Castillo E., Frias E.S., Swanson R.A. (2018). Bioenergetic Regulation of Microglia. Glia.

[86] Joshi A.U., Minhas P.S., Liddelow S.A., Haileselassie B., Andreasson K.I., Dorn G.W., Mochly-Rosen D. (2019). Fragmented Mitochondria Released from Microglia Trigger A1 Astrocytic Response and Propagate Inflammatory Neurodegeneration. Nat. Neurosci..

[87] Zhang Y., Zhao L., Wang X., Ma W., Lazere A., Qian H.-H., Zhang J., Abu-Asab M., Fariss R.N., Roger J.E. (2018). Repopulating Retinal Microglia Restore Endogenous Organization and Function under CX3CL1-CX3CR1 Regulation. Sci. Adv..

[88] Gratuze M., Chen Y., Parhizkar S., Jain N., Strickland M.R., Serrano J.R., Colonna M., Ulrich J.D., Holtzman D.M. (2021). Activated Microglia Mitigate Aβ-Associated Tau Seeding and Spreading. J. Exp. Med..

[89] Sanchez-Mico M.V., Jimenez S., Gomez-Arboledas A., Muñoz-Castro C., Romero-Molina C., Navarro V., Sanchez-Mejias E., Nuñez-Diaz C., Sanchez-Varo R., Galea E. (2021). Amyloid-β Impairs the Phagocytosis of Dystrophic Synapses by Astrocytes in Alzheimer’s Disease. Glia.

[90] Zhang Y., Cui D. (2021). Evolving Models and Tools for Microglial Studies in the Central Nervous System. Neurosci. Bull..

[91] Vainchtein I.D., Molofsky A.V. (2020). Astrocytes and Microglia: In Sickness and in Health. Trends Neurosci..

[92] Michael J., Unger M.S., Poupardin R., Schernthaner P., Mrowetz H., Attems J., Aigner L. (2020). Microglia Depletion Diminishes Key Elements of the Leukotriene Pathway in the Brain of Alzheimer’s Disease Mice. Acta Neuropathol. Commun..

[93] Sosna J., Philipp S., Albay R., Reyes-Ruiz J.M., Baglietto-Vargas D., LaFerla F.M., Glabe C.G. (2018). Early Long-Term Administration of the CSF1R Inhibitor PLX3397 Ablates Microglia and Reduces Accumulation of Intraneuronal Amyloid, Neuritic Plaque Deposition and Pre-Fibrillar Oligomers in 5XFAD Mouse Model of Alzheimer’s Disease. Mol. Neurodegener..

[94] Zhao R., Hu W., Tsai J., Li W., Gan W.-B. (2017). Microglia Limit the Expansion of β-Amyloid Plaques in a Mouse Model of Alzheimer’s Disease. Mol. Neurodegener..

[95] Asai H., Ikezu S., Tsunoda S., Medalla M., Luebke J., Haydar T., Wolozin B., Butovsky O., Kügler S., Ikezu T. (2015). Depletion of Microglia and Inhibition of Exosome Synthesis Halt Tau Propagation. Nat. Neurosci..

[96] Reed-Geaghan E.G., Croxford A.L., Becher B., Landreth G.E. (2020). Plaque-Associated Myeloid Cells Derive from Resident Microglia in an Alzheimer’s Disease Model. J. Exp. Med..

[97] Shukla A.K., McIntyre L.L., Marsh S.E., Schneider C.A., Hoover E.M., Walsh C.M., Lodoen M.B., Blurton-Jones M., Inlay M.A. (2019). CD11a Expression Distinguishes Infiltrating Myeloid Cells from Plaque-Associated Microglia in Alzheimer’s Disease. Glia.

[98] Huang Y., Happonen K.E., Burrola P.G., O’Connor C., Hah N., Huang L., Nimmerjahn A., Lemke G. (2021). Microglia Use TAM Receptors to Detect and Engulf Amyloid β Plaques. Nat. Immunol..

[99] Reiss A.B., Arain H.A., Stecker M.M., Siegart N.M., Kasselman L.J. (2018). Amyloid Toxicity in Alzheimer’s Disease. Rev. Neurosci..

[100] Venegas C., Kumar S., Franklin B.S., Dierkes T., Brinkschulte R., Tejera D., Vieira-Saecker A., Schwartz S., Santarelli F., Kummer M.P. (2017). Microglia-Derived ASC Specks Cross-Seed Amyloid-β in Alzheimer’s Disease. Nature.

[101] Mendes M.S., Le L., Atlas J., Brehm Z., Ladron-de-Guevara A., Matei E., Lamantia C., McCall M.N., Majewska A.K. (2021). The Role of P2Y12 in the Kinetics of Microglial Self-Renewal and Maturation in the Adult Visual Cortex in Vivo. eLife.

[102] Perea J.R., Bolós M., Avila J. (2020). Microglia in Alzheimer’s Disease in the Context of Tau Pathology. Biomolecules.

[103] Bolós M., Perea J.R., Avila J. (2017). Alzheimer’s Disease as an Inflammatory Disease. Biomol. Concepts.

[104] Nagamine S., Yamazaki T., Makioka K., Fujita Y., Ikeda M., Takatama M., Okamoto K., Yokoo H., Ikeda Y. (2016). Hypersialylation Is a Common Feature of Neurofibrillary Tangles and Granulovacuolar Degenerations in Alzheimer’s Disease and Tauopathy Brains. Neuropathology.

[105] Bolós M., Llorens-Martín M., Jurado-Arjona J., Hernández F., Rábano A., Avila J. (2015). Direct Evidence of Internalization of Tau by Microglia In Vitro and In Vivo. J. Alzheimers Dis..

[106] Hopp S.C., Lin Y., Oakley D., Roe A.D., DeVos S.L., Hanlon D., Hyman B.T. (2018). The Role of Microglia in Processing and Spreading of Bioactive Tau Seeds in Alzheimer’s Disease. J. Neuroinflamm..

[107] Luo W., Liu W., Hu X., Hanna M., Caravaca A., Paul S.M. (2015). Microglial Internalization and Degradation of Pathological Tau Is Enhanced by an Anti-Tau Monoclonal Antibody. Sci. Rep..

[108] Andersson C.R., Falsig J., Stavenhagen J.B., Christensen S., Kartberg F., Rosenqvist N., Finsen B., Pedersen J.T. (2019). Antibody-Mediated Clearance of Tau in Primary Mouse Microglial Cultures Requires Fcγ-Receptor Binding and Functional Lysosomes. Sci. Rep..

[109] Dai C., Hu W., Tung Y.C., Liu F., Gong C.-X., Iqbal K. (2018). Tau Passive Immunization Blocks Seeding and Spread of Alzheimer Hyperphosphorylated Tau-Induced Pathology in 3 × Tg-AD Mice. Alzheimers Res. Ther..

[110] Acker C.M., Forest S.K., Zinkowski R., Davies P., d’Abramo C. (2013). Sensitive Quantitative Assays for Tau and Phospho-Tau in Transgenic Mouse Models. Neurobiol. Aging.

[111] Lewis J., McGowan E., Rockwood J., Melrose H., Nacharaju P., Slegtenhorst M.V., Gwinn-Hardy K., Murphy M.P., Baker M., Yu X. (2000). Neurofibrillary Tangles, Amyotrophy and Progressive Motor Disturbance in Mice Expressing Mutant (P301L) Tau Protein. Nat. Genet..

[112] Yoshiyama Y., Higuchi M., Zhang B., Huang S.-M., Iwata N., Saido T.C., Maeda J., Suhara T., Trojanowski J.Q., Lee V.M.-Y. (2007). Synapse Loss and Microglial Activation Precede Tangles in a P301S Tauopathy Mouse Model. Neuron.

[113] Ramsden M., Kotilinek L., Forster C., Paulson J., McGowan E., SantaCruz K., Guimaraes A., Yue M., Lewis J., Carlson G. (2005). Age-Dependent Neurofibrillary Tangle Formation, Neuron Loss, and Memory Impairment in a Mouse Model of Human Tauopathy (P301L). J. Neurosci..

[114] Bemiller S.M., McCray T.J., Allan K., Formica S.V., Xu G., Wilson G., Kokiko-Cochran O.N., Crish S.D., Lasagna-Reeves C.A., Ransohoff R.M. (2017). TREM2 Deficiency Exacerbates Tau Pathology through Dysregulated Kinase Signaling in a Mouse Model of Tauopathy. Mol. Neurodegener..

[115] Bhaskar K., Konerth M., Kokiko-Cochran O.N., Cardona A., Ransohoff R.M., Lamb B.T. (2010). Regulation of Tau Pathology by the Microglial Fractalkine Receptor. Neuron.

[116] Maphis N., Xu G., Kokiko-Cochran O.N., Jiang S., Cardona A., Ransohoff R.M., Lamb B.T., Bhaskar K. (2015). Reactive Microglia Drive Tau Pathology and Contribute to the Spreading of Pathological Tau in the Brain. Brain J. Neurol..

[117] Acharya M.M., Green K.N., Allen B.D., Najafi A.R., Syage A., Minasyan H., Le M.T., Kawashita T., Giedzinski E., Parihar V.K. (2016). Elimination of Microglia Improves Cognitive Function Following Cranial Irradiation. Sci. Rep..

[118] Li M., Li Z., Ren H., Jin W.-N., Wood K., Liu Q., Sheth K.N., Shi F.-D. (2017). Colony Stimulating Factor 1 Receptor Inhibition Eliminates Microglia and Attenuates Brain Injury after Intracerebral Hemorrhage. J. Cereb. Blood Flow Metab. Off. J. Int. Soc. Cereb. Blood Flow Metab..

[119] Jin W.-N., Shi S.X.-Y., Li Z., Li M., Wood K., Gonzales R.J., Liu Q. (2017). Depletion of Microglia Exacerbates Postischemic Inflammation and Brain Injury. J. Cereb. Blood Flow Metab. Off. J. Int. Soc. Cereb. Blood Flow Metab..

[120] Szalay G., Martinecz B., Lénárt N., Környei Z., Orsolits B., Judák L., Császár E., Fekete R., West B.L., Katona G. (2016). Microglia Protect against Brain Injury and Their Selective Elimination Dysregulates Neuronal Network Activity after Stroke. Nat. Commun..

[121] Butowski N., Colman H., De Groot J.F., Omuro A.M., Nayak L., Wen P.Y., Cloughesy T.F., Marimuthu A., Haidar S., Perry A. (2016). Orally Administered Colony Stimulating Factor 1 Receptor Inhibitor PLX3397 in Recurrent Glioblastoma: An Ivy Foundation Early Phase Clinical Trials Consortium Phase II Study. Neuro-Oncology.

[122] Streit W.J., Khoshbouei H., Bechmann I. (2020). Dystrophic Microglia in Late-Onset Alzheimer’s Disease. Glia.

[123] Wang S., Mustafa M., Yuede C.M., Salazar S.V., Kong P., Long H., Ward M., Siddiqui O., Paul R., Gilfillan S. (2020). Anti-Human TREM2 Induces Microglia Proliferation and Reduces Pathology in an Alzheimer’s Disease Model. J. Exp. Med..

[124] Sevigny J., Chiao P., Bussière T., Weinreb P.H., Williams L., Maier M., Dunstan R., Salloway S., Chen T., Ling Y. (2016). The Antibody Aducanumab Reduces Aβ Plaques in Alzheimer’s Disease. Nature.

[125] Ostrowitzki S., Deptula D., Thurfjell L., Barkhof F., Bohrmann B., Brooks D.J., Klunk W.E., Ashford E., Yoo K., Xu Z.X. (2012). Mechanism of Amyloid Removal in Patients with Alzheimer Disease Treated with Gantenerumab. Arch. Neurol..

[126] Bohrmann B., Baumann K., Benz J., Gerber F., Huber W., Knoflach F., Messer J., Oroszlan K., Rauchenberger R., Richter W.F. (2012). Gantenerumab: A Novel Human Anti-Aβ Antibody Demonstrates Sustained Cerebral Amyloid-β Binding and Elicits Cell-Mediated Removal of Human Amyloid-β. J. Alzheimers Dis..

[127] Novakovic D., Feligioni M., Scaccianoce S., Caruso A., Piccinin S., Schepisi C., Errico F., Mercuri N.B., Nicoletti F., Nisticò R. (2013). Profile of Gantenerumab and Its Potential in the Treatment of Alzheimer’s Disease. Drug Des. Dev. Ther..

