# Supplementary material for: Should We Open Fire on Microglia? Depletion Models as Tools to Elucidate Microglial Role in Health and Alzheimer’s Disease
Source: Int J Mol Sci. 2021 Sep 8;22(18):9734. doi: 10.3390/ijms22189734 (PMC8471219; doi:10.3390/ijms22189734)
Supplement: Supplementary file 1 [file ijms-22-09734-s001.zip › ijms-1336685-supplementary.pdf]

Supplementary Materials

Table S1. Antibodies and Taqman Gene Expression assay probes (Applied Biosystems) used. Flow cytometry analysis was developed as described in March-Diaz et al., 2021 [18]. Immunohistochemistry and qPCR was performed as described in Romero-Molina et al., 2018 [20].

	Antibody	Clonality	Vendor	Dilution
**Flow cytometry**	Anti-CD45-PE	Monoclonal (Clone HI30)	Immunostep	1/33
	Anti-CD11b	Monoclonal (Clone M1/70)	Inmunostep	1/33
**Immunohistochemistry**	Anti-Iba1	Polyclonal (#234 003)	Synaptic Systems	1/1000

	Taqman Gene Expression Assay Probe	Reference
**qPCR**	*Cd163*	Mm00474091_m1
	Cd3	Mm01179194_m1
	Cd45	Mm01293577_m1
	Ccr2	Mm00801681_m1
	Clec7a	Mm01183349_m1
	Ki67	Mm01278617_m1
	Lgals3	Mm00802901_m1
	Ly6c	Mm00841873_m1
	Trem2	Mm04209424_g1
